# Global information-seeking behavior of air pollution and cardiovascular disease: insights from google trends analysis

**DOI:** 10.3389/fepid.2026.1874654

**Published:** 2026-07-08

**Authors:** Javeria Akhter, Talha Ali, Aasiya Shahbaz, Noor Ul Ain Shahzad, Jamil Nasrallah

**Affiliations:** 1 Lung Health Program, Community Health Directorate, Indus Hospital and Health Network, Karachi, Pakistan; 2 Department of Medicine, Shaheed Mohtarma Benazir Bhutto Medical College Lyari, Karachi, Pakistan; 3 Department of Medicine, Dow University of Health Sciences, Karachi, Pakistan; 4 Department of Medicine, Faculty of Medical Sciences, Lebanese University, Beirut, Lebanon

**Keywords:** air pollution, cardiovascular disease, google trends, infodemiology, information-seeking behavior

## Abstract

**Introduction:**

Air pollution (AP) contributes to over 4.2 million premature deaths annually, with approximately 20% linked to cardiovascular disease (CVD)-related deaths. Despite robust scientific evidence, information-seeking behavior remains poorly characterized. This study applies an infodemiological approach to assess global information-seeking behavior related to AP and its cardiovascular implications.

**Methods:**

A retrospective analysis was conducted using Google Trends data from June 2020 to June 2025. Five AP-related search terms (e.g., “air pollution,” “PM₂.₅,” “air quality index,” “environmental pollution,” and “mask”) and five CVD-related search terms (e.g., “cardiovascular disease”, “heart disease,” “heart attack,” “chest pain,” and “high blood pressure”) were analyzed globally. Pearson correlation coefficients, partial correlation analysis controlling for pandemic-related confounding, and time-series approaches including ARIMAX modeling and Granger causality testing were applied to assess temporal and correlational patterns in information-seeking behavior.

**Results:**

Global information-seeking behavior showed marked variability across AP and CVD search terms. AP showed moderate positive correlations with “CVD”, “heart disease”, and “high blood pressure” (r = 0.275;0.295;0.386, *p* < 0.01). “Environmental pollution” showed a moderate correlation with “heart disease” (r = 0.417, *p* < 0.01) and “blood pressure” (r = 0.315, *p* < 0.01). Technical AP indicators showed weak or negative zero-order associations with most CVD terms; however, sensitivity analysis controlling for pandemic-related confounding revealed complete directional reversal for PM₂.₅, with partial correlations becoming significantly positive, indicating that original negative associations were pandemic-driven artefacts. Search volumes were highest for Chest Pain and High Blood Pressure, with low global engagement for PM₂.₅ and AQI. Twenty-seven countries, including Pakistan, India, and Australia, demonstrated concurrent AP and CVD information-seeking behavior.

**Conclusion:**

This analysis indicates moderate information-seeking behavior in the association between AP and CVD, with geographic variation. Findings highlight the need for targeted public health communication strategies that translate AP risk into accessible cardiovascular health messages.

## 1 Introduction

Air pollution (AP) is recognized as one of the most significant environmental threats to public health, contributing to approximately 4.2 million premature deaths annually and ranking as the fourth leading risk factor for global mortality (1–3). Approximately one in five cardiovascular disease(CVD)-related deaths is attributed to AP exposure, with the Global Burden of Disease (GBD) study estimating 2.46 million CVD deaths and 58.3 million disability-adjusted life years attributable to AP in 2021 (2, 4, 5).

AP encompasses a complex mixture of fine and coarse particulate matter. Among these pollutants, PM₂.₅ is particularly harmful because of its small size, which allows it to penetrate deep into the lungs and enter the bloodstream (6). Such exposure induces oxidative stress and systemic inflammation, contributing to endothelial dysfunction, atherosclerosis, and subsequent cardiovascular events (7, 8, 11). However, despite the extensive evidence linking AP to CVD, information-seeking behavior regarding the AP–CVD relationship remains poorly characterized, highlighting the need for population-level surveillance of health information-seeking behavior (9).

Google Trends is a publicly available tool developed by Google that provides normalized relative search volume (RSV) data for user queries submitted through the Google search engine. Unlike absolute search counts, RSV values reflect proportional indices of interest, allowing targeted extraction of search activity at global, national, and subnational levels. Google Trends has been widely applied in infodemiology — the study of health information patterns in populations — to monitor disease outbreaks, assess information-seeking behavior of health conditions, and identify gaps in health literacy (10, 11). Its utility as a low-cost, real-time proxy for population-level interest has been demonstrated across diverse health domains including infectious diseases, chronic conditions, and environmental health. However, its application examining information-seeking behavior of the AP–CVD relationship remains limited, representing a gap this study addresses (10).

Most prior studies have evaluated search activity for single conditions without linking environmental exposures to their downstream CVD consequences. Furthermore, few studies have applied time-series and lag-based analytical approaches, such as ARIMAX modeling and Granger causality, to examine temporal relationships between AP and CVD-related information-seeking behavior. Identifying such gaps can help guide targeted risk communication, strengthen prevention strategies, and inform public health practitioners in designing interventions that link environmental exposures with cardiovascular risk.

This study analyses five years of global Google Trends data (June 2020–June 2025) to evaluate whether information-seeking behavior related to AP is associated with CVD-related search activity, using infodemiological and time-series analytical approaches.

## 2 Methods

### 2.1 Data sources

Google Trends provides normalized RSV data ranging from 0 to 100, where 100 denotes peak search interest within the selected time frame and geography. Only terms meeting a minimum search activity threshold are included in results.

### 2.2 Study design and framework

This study was conducted based on the methodological framework developed by Mavragani and Ochoa, which outlines procedures for selecting appropriate regions and time frames to retrieve and interpret Google Trends data (11).

### 2.3 Operational definitions

#### 2.3.1 Air pollution

AP was operationally defined as ambient environmental pollution resulting from chemical and particulate exposures, aligning with the selected search terms.

#### 2.3.2 Cardiovascular disease

CVD was operationally defined to include non-communicable conditions affecting the heart and blood vessels, including ischemic heart disease, hypertension, and acute coronary syndromes, as reflected in the selected search terms.

Although stroke is commonly classified as CVD, it was excluded as lay searches for stroke-related terms tend to reflect acute symptom recognition rather than chronic cardiovascular risk awareness, which is beyond the cardiac-focused scope of this analysis.

### 2.4 Ethical approval

The study exclusively utilized publicly available and anonymized data from Google Trends. As no human participants were involved and no identifiable personal data were collected institutional review board approval and informed consent were not required.

### 2.5 Search strategy and data collection

Google Trends data were analyzed for the period June 2020 to June 2025, with the geographic setting limited to Worldwide, category set to All Categories, and search type set to Web Search. Ten keywords were grouped into two categories: five AP-related terms (“air pollution,” “environmental pollution,” “PM₂.₅,” “air quality index,” and “mask”) and five CVD-related terms (“cardiovascular disease,” “heart disease,” “heart attack,” “high blood pressure,” and “chest pain”). Keywords were selected based on three criteria: relevance to established AP and CVD literature, prior use in infodemiological and Google Trends studies, and likelihood of use by the general public rather than healthcare professionals (12–15).

The search was restricted to English-language terms to ensure cross-country comparability within a single Google Trends query session, as multi-language queries cannot be directly compared within the same scaling framework. Several related terms were excluded — including “smog,” “air quality,” “hypertension,” and “cardiac arrest” — to avoid query-scaling distortions and unrelated search contexts. The term “mask” was included as a proxy for airborne exposure protection but was substantially confounded by COVID-19-related search behavior during the study period and is interpreted accordingly. “Chest pain” was included as a cardinal symptom of acute coronary syndromes; however, as a non-specific symptom with multiple potential causes.

### 2.6 Data extraction

Data were extracted from Google Trends on 1 July 2025 using the Search Term option. All keywords were entered manually within the same grouped query session using identical filters — worldwide, web search, all categories, June 2020 to June 2025 — to maintain consistent scaling across all terms simultaneously. RSV values were exported directly in CSV format from the Google Trends Explore interface.

As Google Trends normalizes scores relative to peak search volume within each query session, direct cross-country comparisons should be interpreted with caution. Inter-regional normalization beyond session-level consistency was not performed. To assess robustness of estimates, data extraction was repeated on two separate occasions, with consistent patterns observed and no meaningful variation in trend direction or correlation estimates.

For regional analysis, Countries were included in the geographic analysis if they met two criteria: first, availability of RSV data for at least one AP-related and one CVD-related search term; and second, non-zero RSV values across a minimum number of time points sufficient for meaningful comparison. Countries with consistently missing or zero RSV values across all keywords were excluded from country-level comparisons, as such values indicate insufficient search volume to meet Google Trends' minimum reporting threshold.

### 2.7 Data analysis

Data were cleaned and processed using SPSS software (version 26). Missing or zero RSV values were treated as indicative of low or insufficient information-seeking behavior rather than true absence of interest, and no imputation was performed.

For regional analysis, countries were included if they met two criteria: first, availability of RSV data for at least three AP-related and three CVD-related search term; and second, combined RSV sum exceeding 50 across all search term.

Pearson correlation coefficients were computed to assess associations between AP and CVD search terms. Correlation strength was interpreted using standard thresholds: 0.00–0.19 very weak, 0.20–0.39 weak, 0.40–0.59 moderate, 0.60–0.79 strong, and ≥0.80 very strong. As a sensitivity analysis, partial correlation analysis was conducted controlling for “mask” to assess the influence of COVID-19 pandemic confounding on observed AP–CVD associations.

Time-series analyses were conducted using R (version 4.5.1) on monthly data. Standardized indices for AP (AP_Z) and CVD (CVD_Z) were constructed along with lag variables (lag −2 to +2). An autoregressive integrated moving average model with exogenous variables (ARIMAX) was applied to assess the association between AP and CVD information-seeking behavior while accounting for temporal autocorrelation. Model performance was evaluated using Akaike Information Criterion (AIC), root mean square error (RMSE), and autocorrelation function (ACF) of residuals. Lagged associations were examined across lag structures (lag 0, lag 1, and lag 2), with model selection based on AIC comparison.

Bidirectional Granger causality tests were performed using the “lmtest” package in R to evaluate whether AP information-seeking behavior predicts CVD trends and vice versa, with a lag order of 2 selected based on model fit criteria. Statistical significance was defined as *p* < 0.05.

### 2.8 Data output

**Figure 1:**
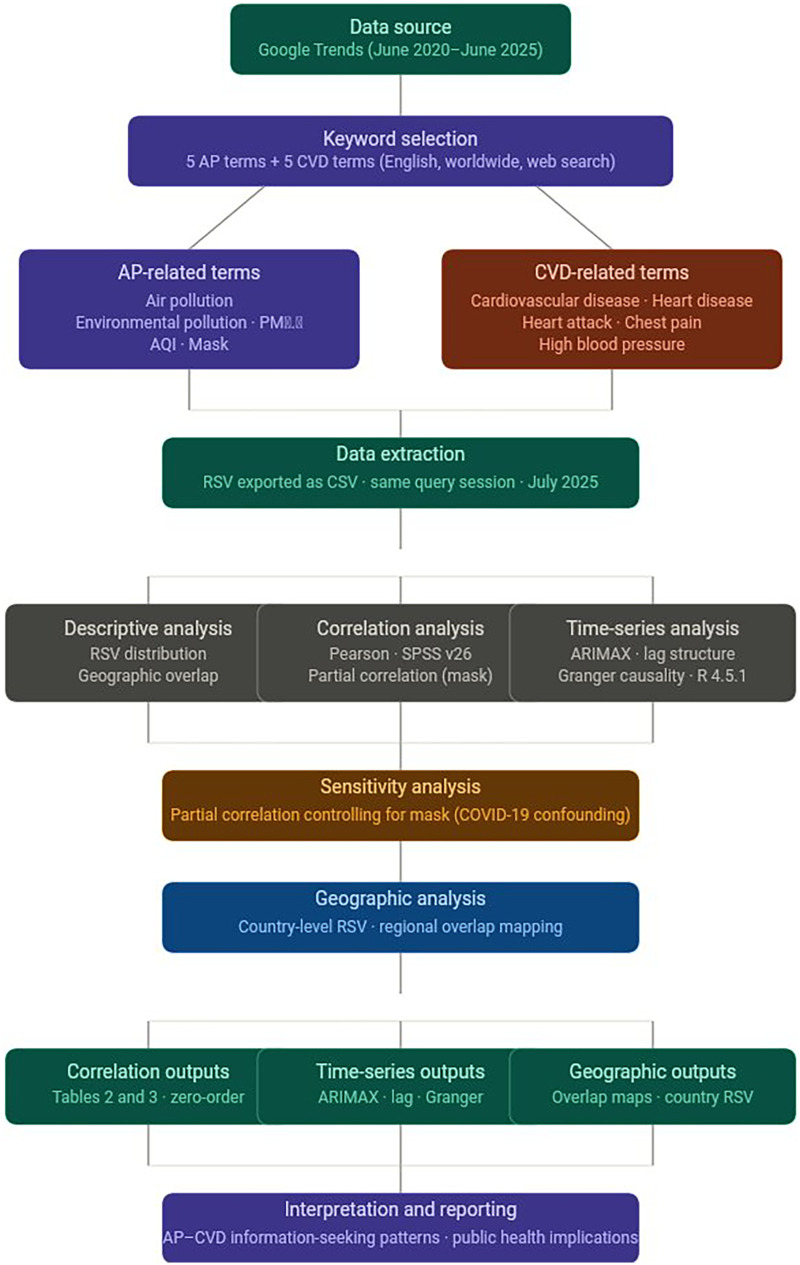
Methodological workflow diagram illustrating the sequential analytical stages of the study.

Results were summarized through tables and graphical visualizations illustrating the temporal distribution and geographic concentration of information-seeking behavior. Outputs included correlation matrices, country-level RSV rankings, choropleth maps depicting geographic overlap, a frequency-based word cloud reflecting keyword distribution patterns, ARIMAX model statistics including regression coefficients and model fit indices, lag-effect visualizations, and Granger causality results (Figure 1).

## 3 Results

### 3.1 Descriptive analysis of information-seeking behavior

The analysis of global search volumes revealed marked variability in information-seeking behavior across AP and CVD search terms (Table 1).

**Table 1:** Frequency of Air pollution and cardiovascular disease–related terms by relative search volume (RSV) > 50 across countries, June 2020– June 2025.

Relative Search Volume	Frequency
Air Pollution	4 (1.6%)
Environmental Pollution	1 (0.4%)
PM_2.5_	4 (1.6%)
Air Quality Index	2 (0.8%)
Mask	16 (6.4%)
Cardiovascular Disease	15 (6%)
Heart Disease	4 (1.6%)
Heart Attack	4 (1.6%)
High Blood Pressure	15 (6%)
Chest Pain	11 (4.4%)

**Figure 2:**
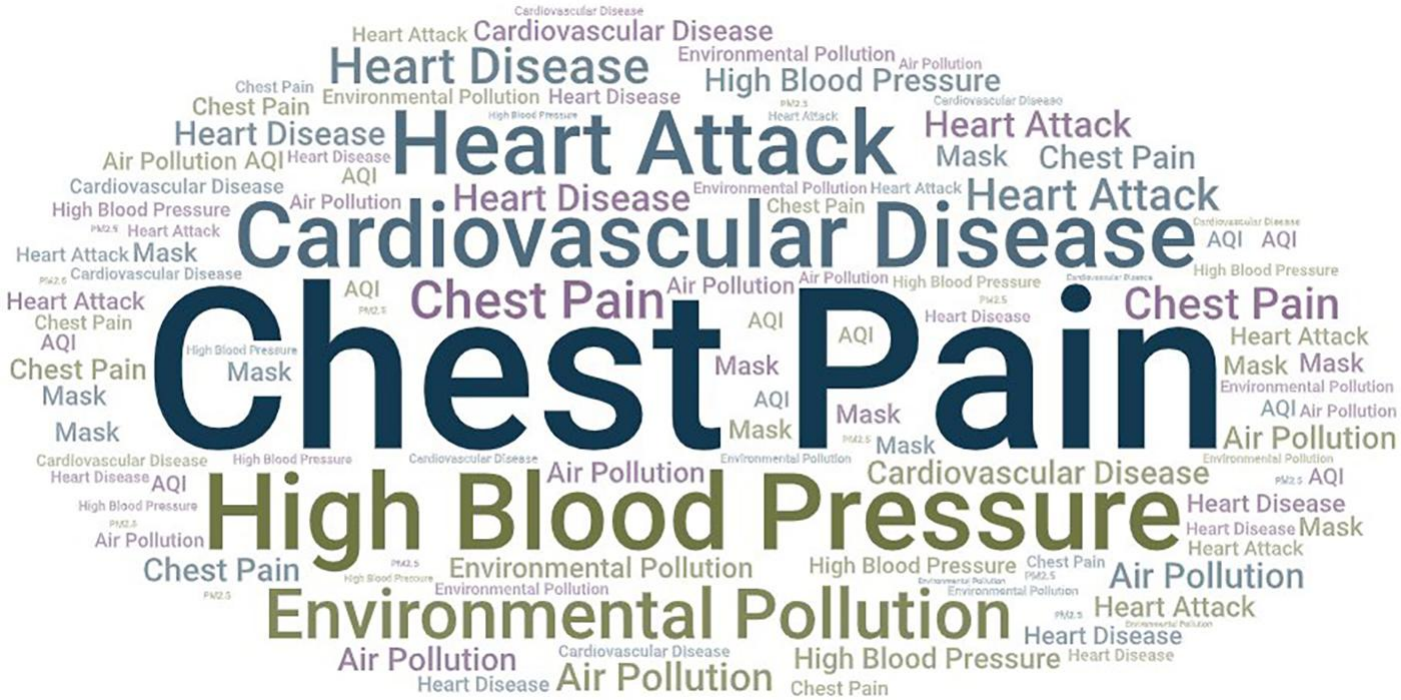
Word cloud of AP and CVD-related search terms weighted by mean relative search volume across the study period (June 2020–June 2025).

Figure 2 presents a word cloud in which font size is proportional to mean RSV across the study period (June 2020–June 2025). Symptom-based CVD terms dominated global information-seeking behavior, particularly Chest Pain (mean RSV=81), High Blood Pressure (mean RSV=80), and CVD (mean RSV=72). In contrast, technical AP indicators showed the lowest mean RSV values, with PM₂.₅ (mean RSV=18) and AQI (mean RSV=7) appearing smallest, consistent with limited global engagement with technical pollution metrics.

### 3.2 Geographic distribution

**Figure 3:**
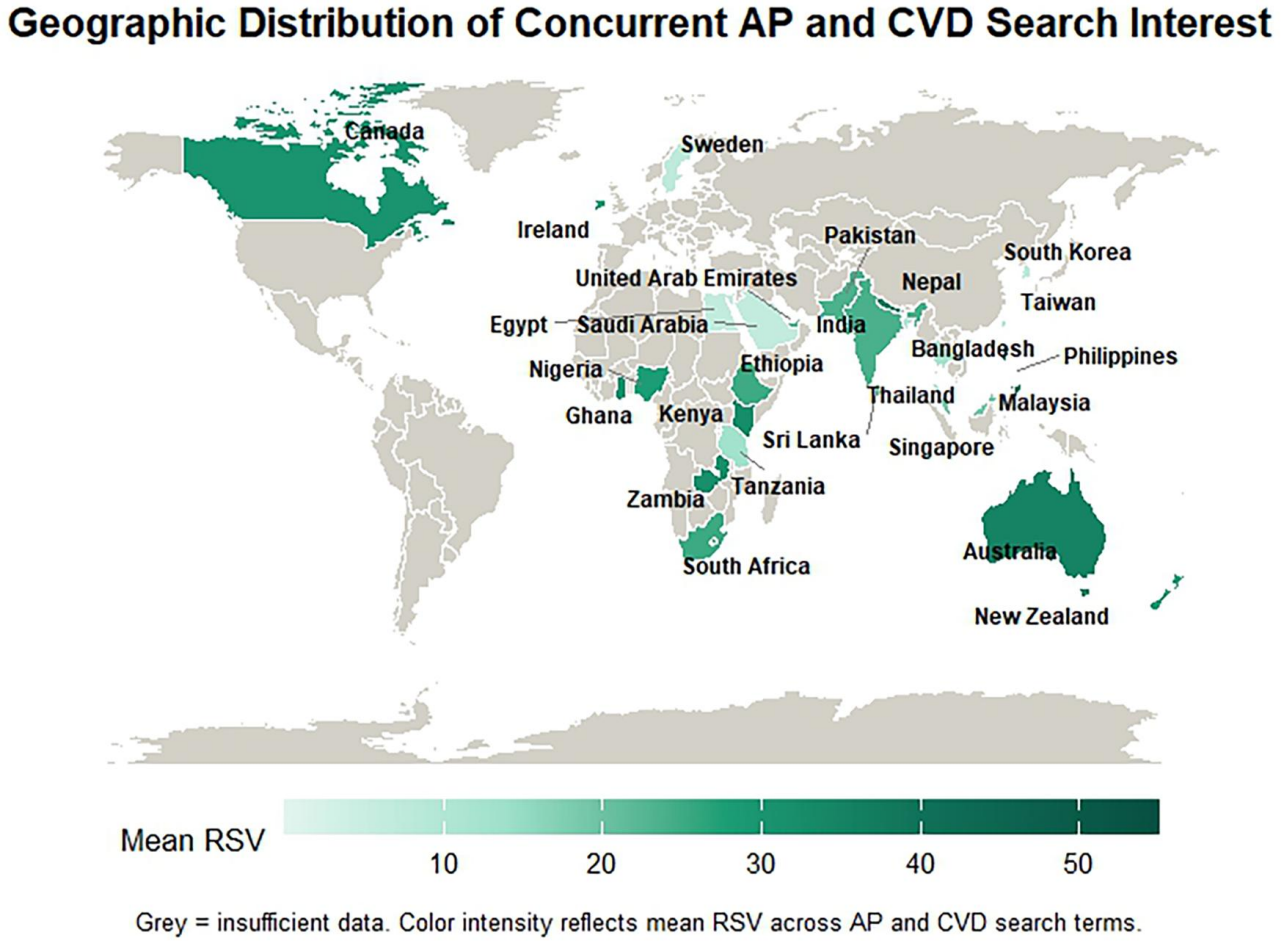
Choropleth map depicting geographic distribution of concurrent AP and CVD information-seeking behavior across 27 countries (June 2020–June 2025).

Geographic distribution of concurrent AP and CVD information-seeking behavior across 27 countries is presented in Figure 3. Color intensity reflects the combined RSV sum across all AP and CVD search terms, with darker shading indicating higher concurrent information-seeking behavior. The highest combined RSV values were observed in South Asian countries — particularly Nepal (combined RSV=340) and Philippines (combined RSV=405) — alongside Sub-Saharan African nations including Ghana (combined RSV=316) and Kenya (combined RSV=322), and high-income countries including Australia (combined RSV=351), Canada (combined RSV=304), and Ireland (combined RSV=280). Countries shown in grey had insufficient search engagement across both AP and CVD domains, failing to meet the inclusion threshold of combined RSV exceeding 50 with non-zero values across a minimum of three search terms. (Supplementary Table S1).

City-level analysis highlighted geographic clustering of peak information-seeking behavior in major urban centers across South Asia, North America, and Sub-Saharan Africa. (Supplementary Figure S1).

### 3.3 Correlation analysis

Pearson correlation analysis revealed moderate positive associations between general AP and CVD-related search terms (r = 0.29–0.42, *p* < 0.01). Air Pollution demonstrated significant positive correlations with CVD (r = 0.386), heart disease (r = 0.295), and High Blood Pressure (r = 0.275), while Environmental Pollution showed moderate correlations with heart disease (r = 0.417) and Blood Pressure (r = 0.315) (all *p* < 0.01). Technical AP indicators — PM₂.₅ and AQI — showed weak or negative correlations with most CVD-related search terms. The term “Mask” showed a strong positive correlation with Chest Pain (r = 0.598, *p* < 0.01), likely reflecting concurrent COVID-19-driven search behavior rather than AP-related cardiovascular information-seeking (Table 2).

**Table 2:** Pearson correlation coefficients for Air pollution and CVD-related search terms in google trends data (June 2020– June 2025).

Air Pollution			
CVD Term	**Correlation**	**Significance**	**Interpretation**
CVD	.386**	*p* < 0.01	Moderate positive correlation
Heart Disease	.295**	*p* < 0.01	Moderate, weaker than with general CVD
Heart Attack	.095	Not significant	No significant association
Chest Pain	-.101	Not significant	Slight negative but not significant
Blood Pressure	.275**	*p* < 0.01	Moderate positive — may reflect risk awareness
Environmental Pollution			
CVD	.370**	*p* < 0.01	Moderate positive
Heart Disease	.417**	*p* < 0.01	Slightly stronger than with AP
Heart Attack	.252**	*p* < 0.01	Weak–moderate positive
Chest Pain	-.097	Not significant	Not significantly associated
Blood Pressure	.315**	*p* < 0.01	Moderate positive
PM_2.5_			
CVD	-.139*	*p* < 0.05	Weak negative
Heart Disease	-.066	Not significant	No association
Heart Attack	-.175**	*p* < 0.01	Weak inverse correlation
Chest Pain	.403**	*p* < 0.01	Strong positive — surprising
Blood Pressure	-.085	Not significant	Weak negative
AQI			
CVD	-.148*	*p* < 0.05	Weak negative
Heart Disease	-.092	Not significant	No meaningful link
Heart Attack	-.121*	*p* < 0.05	Weak negative
Chest Pain	-.107	Not significant	Slight inverse
Blood Pressure	-.218**	*p* < 0.01	Weak–moderate negative
Mask			
CVD	-.430**	*p* < 0.01	Moderate negative
Heart Disease	-.236**	*p* < 0.01	Weak–moderate negative
Heart Attack	-.195**	*p* < 0.01	Weak negative
Chest Pain	.598**	*p* < 0.01	Strong positive — likely reflects COVID searches
Blood Pressure	-.304**	*p* < 0.01	Moderate negative

*,** denotes the significance of the association, in either the positive or negative direction.

As a sensitivity analysis, partial correlation analysis controlling for “mask” as a proxy for COVID-19 pandemic-related search behavior revealed three distinct patterns (Table 3). First, general AP terms — Air Pollution and Environmental Pollution — showed modest attenuation but remained significantly positively associated with CVD, Heart Disease, and Blood Pressure, confirming robustness of these associations independent of pandemic confounding. Second, PM₂.₅ demonstrated complete directional reversal; originally negative zero-order correlations with CVD (r = −0.139), heart disease (r = −0.066), and Blood Pressure (r = −0.085) became significant positive partial correlations (r = 0.318, r = 0.180, and r = 0.236 respectively, all *p* < 0.01), indicating these original associations were pandemic-driven artefacts. The PM₂.₅–Chest Pain association similarly reversed from r = 0.403 (*p* < 0.001) to r = −0.097 (ns). Third, Chest Pain associations with general AP terms attenuated to non-significance after controlling for mask, confirming pandemic-driven distortion. Notably, AQI–Chest Pain strengthened negatively from r = −0.107 (ns) to r = −0.221 (*p* < 0.001), suggesting an independent negative relationship between technical air quality searches and symptom-driven cardiovascular information-seeking when pandemic influence is removed.

**Table 3:** Zero-Order and partial correlation coefficients between Air pollution and cardiovascular disease search terms, controlling for “Mask".

AP Term	CVD Term	r (With mask)	r (Without mask)	Change
Air Pollution	CVD	0.386	0.342**	Slightly attenuated, significant
Air Pollution	Heart Disease	0.295	0.261**	Slightly attenuated, significant
Air Pollution	Heart Attack	0.095	0.072	Attenuated, non-significant
Air Pollution	Chest Pain	−1.01	0.016	Sign reversal, non-significan
Air Pollution	Blood Pressure	0.275	0.229**	Slightly attenuated, significant
Environmental Pollution	CVD	0.370	0.331**	Slightly attenuated, significant
Environmental Pollution	Heart Disease	0.417	0.392**	Minimal change, significant
Environmental Pollution	Heart Attack	0.252	0.234**	Slightly attenuated, significant
Environmental Pollution	Chest Pain	-.097	0.008	Sign reversal, non-significant
Environmental Pollution	Blood Pressure	0.315	0.279**	Slightly attenuated, significant
AQI	CVD	-.148	-.109	Attenuated to non-significant
AQI	Heart Disease	-.092	-.067	Both non-significant, stable
AQI	Heart Attack	-.121	-.100	Both non-significant, stable
AQI	Chest Pain	-.107	-.221**	Strengthened, significant
AQI	Blood Pressure	-.218	-.194**	Slightly attenuated, significant
PM_2.5_	CVD	-.139	0.318**	Complete reversal
PM_2.5_	Heart Disease	-.066	0.180**	Complete reversal
PM_2.5_	Heart Attack	-.175	−0.045	Attenuated to non-significant
PM_2.5_	Chest Pain	0.403	−0.097**	Complete reversal
PM_2.5_	Blood Pressure	-.085	0.236**	Complete reversal

*p < 0.05, **p < 0.01. r (With mask) = zero-order Pearson correlation; r (Without mask) = partial correlation controlling for mask as a proxy for COVID-19 pandemic-related search behaviour. Positive values indicate co-directional search interest; negative values indicate inverse search patterns.

### 3.4 Time-Series analysis

To further explore temporal dynamics beyond correlation, time-series analyses were conducted using monthly RSV data.

#### 3.4.1 ARIMAX model results

The ARIMAX model demonstrated a statistically significant positive association between AP and CVD information-seeking behavior (*β*=0.218, SE = 0.096), indicating that higher AP-related information-seeking behavior was associated with increased CVD-related search activity. Model diagnostics indicated acceptable residual independence (ACF1 = 0.067), with an AIC of 146.97 and moderate predictive error (RMSE=0.79).

#### 3.4.2 Lag structure analysis

Comparison of ARIMAX models across lag structures revealed that the lag-2 model provided the best fit (AIC=141.96), compared to lag-0 (AIC=146.97) and lag-1 (AIC=149.82), suggesting that the strongest association between AP and CVD information-seeking behavior occurred after a two-month delay. Lag-effect visualization confirmed a peak correlation at lag 2, supporting the model-based findings and indicating that increases in AP-related information-seeking behavior may precede cardiovascular-related search activity (Supplementary Figure S3).

#### 3.4.3 Granger causality analysis

Bidirectional Granger causality testing revealed no statistically significant predictive relationship between AP and CVD information-seeking behavior in either direction (AP→CVD: F = 0.653, *p* = 0.524; CVD→AP: F = 0.402, *p* = 0.671).

Overall, findings suggest modest temporal alignment between AP and CVD information-seeking behavior, with evidence of a delayed association but no predictive causality. (Supplementary Figure S2).

## 4 Discussion

This worldwide Google Trends analysis suggests a potential gap between the scientific consensus on AP as a leading risk factor for CVD and global online information-seeking behavior related to this association. Despite extensive evidence confirming that long-term AP exposure increases CVD morbidity and mortality, information-seeking behavior for technical AP indicators — including “Air Pollution,” “PM₂.₅,” and “AQI” — was low in more than 89% of countries. In 2021, the Global Burden of Disease study reported approximately 2.46 million CVD deaths and 58.3 million disability-adjusted life years attributable to AP, underscoring the need to translate exposure science into clear and actionable health messages (2).

Time-series analysis provided additional insights into temporal dynamics between AP and CVD information-seeking behavior. The ARIMAX model demonstrated a modest but statistically significant positive association, while lag analysis indicated that peak associations occurred after a two-month delay, suggesting that increases in AP-related information-seeking behavior may precede cardiovascular-related search activity. However, Granger causality analysis did not demonstrate a predictive relationship in either direction, indicating that while temporal associations exist, they do not imply directional causality. Temporal patterns may additionally be influenced by broader changes in healthcare delivery and public health priorities — for instance, greater emphasis on hypertension screening, cardiovascular risk stratification, and telemedicine may have enhanced engagement with general cardiovascular search terms, whereas environmental risk factors such as AP remain less frequently integrated into routine clinical communication and diagnostic frameworks (16).

General health terms including “Mask,” “CVD,” and “High Blood Pressure” showed relatively greater engagement, suggesting that the public is more responsive to immediate and personally salient health prompts than to environmental exposures that are less visible or less directly perceived. The concurrent high search volumes and positive correlation between “Mask” and “Chest Pain” likely reflect acute symptom surveillance during the pandemic rather than improved understanding of pollution-driven CVD risk (17–21). Sensitivity analysis using partial correlation confirmed that pandemic confounding was most pronounced for PM₂.₅ and Chest Pain associations, while general AP term associations with CVD information-seeking behaviour remained robust after controlling for mask. The complete directional reversal of PM₂.₅ correlations after removing pandemic influence suggests that technical AP indicators may have greater public salience than zero-order analyses initially implied. This pattern likely reflects infodemic dynamics, whereby high-volume rapidly spreading search terms often referred to as “infodemic monikers” distort typical information-seeking behavior. During COVID-19, heightened media coverage and public concern about respiratory symptoms and protective measures likely drove simultaneous spikes in searches for “mask” and symptom-related terms such as “chest pain,” independent of any direct epidemiological relationship (22).

Additionally, this interpretation aligns with broader trends in digital health during COVID-19, where resources and innovation were directed toward immediate needs — including diagnostics, acute telemedicine, contact tracing, and population surveillance — while longer-term priorities for chronic disease prevention and self-management received comparatively less attention (23–26).

The concurrent information-seeking behavior for AP and CVD terms across 27 countries spanning high-, middle-, and low-income regions represents a valuable signal in digital epidemiology, potentially reflecting populations where information-seeking behavior is already primed for targeted interventions. The moderate positive correlations between general AP terms and CVD-related searches suggest that public concern may be partially aligning with epidemiological risk in some settings. In contrast, weaker or negative correlations for technical metrics such as PM₂.₅ and AQI indicate limited public connection between these indicators and personal health outcomes, reflecting broader challenges in environmental health communication (27, 28).

This gap is notable when considered alongside epidemiological evidence. A 2020 meta-analysis reported that each 10 µg/m^3^ increase in long-term PM₂.₅ exposure was associated with a 23% higher risk of ischemic heart disease mortality, a 24% higher risk of cerebrovascular mortality, and modestly increased risks of incident stroke (13%) and myocardial infarction (8%) (29). Despite these robust associations, PM₂.₅ metrics are seldom incorporated into public health messaging or clinical prevention strategies. Supervia et al. (30) demonstrated that even among patients with CVD, understanding of AP indicators was limited, though targeted educational interventions significantly improved information-seeking behavior, highlighting the gap between environmental risk evidence and practical CVD prevention efforts (30).

The observed gap between AP and CVD information-seeking behavior highlights the need for targeted public health strategies. The cardiovascular effects of AP are preventable through policy, urban planning, and behavioral change at the population level, but only if the public perceives AP as harmful (31). Enhanced air quality standards, emission reductions, and improved urban infrastructure can reduce AP exposure and consequently lower CVD risk (32–34). Urban planning measures including improved road design and expanded green spaces can further reduce exposure in high-risk densely populated cities (32), while population-level behavioural changes — such as adopting diets rich in flavonoids and antioxidants and restricting outdoor exposure during high-pollution events — may additionally mitigate cardiovascular risk (31, 35–37). Targeted interventions among high-risk groups including children, older adults, and individuals with pre-existing CVD — who stand to benefit most from symptom-focused messaging — are also recommended (27, 30, 36, 38, 39). Practical preventive measures such as indoor air filtration systems, high-efficiency masks during high-pollution episodes, and real-time air quality monitoring have demonstrated potential to mitigate both short- and long-term cardiovascular risks when combined with policy-level actions (40).

Digital tools represent a significant opportunity to bridge this gap. Systems that combine real-time air quality data with symptom reporting can translate technical AP indicators into personally relevant health information (41, 42). Communicating AP risk through concrete cardiovascular experiences — such as chest pain, shortness of breath, or blood pressure changes — is likely more effective than conveying abstract metrics such as particulate matter concentration (43–45). Notably, recent forecasts indicate that unless targeted interventions are implemented, AP-related CVD mortality and DALYs will increase substantially by 2050 in low- and middle-sociodemographic index countries, rendering public engagement not only desirable but imperative.

Bridging the information-seeking behavior gap requires communication strategies that translate technical AP indicators — such as PM₂.₅ and AQI — into tangible cardiovascular health outcomes including chest pain, hypertension, and heart attack. Policies to improve urban air quality should be paired with public education campaigns to strengthen population-level CVD prevention. The observed correlations in this study reflect population-level information-seeking behavior rather than confirmed epidemiological relationships, and should not be interpreted as direct measures of disease prevalence, incidence, or environmental exposure, as Google Trends RSV values are influenced by media attention, public health campaigns, and concurrent global events.

## 5 Limitations

Several important limitations of this analysis should be acknowledged. First, Google Trends data are subject to biases related to internet penetration, language use, and search engine preference, and may only partially reflect offline information-seeking behavior. As Google is not the dominant search platform in all regions — with Baidu, Yandex, and Naver predominating in China, Russia, and South Korea respectively — variations in search engine preference may affect the representativeness and comparability of findings across settings.

Second, Google Trends provides normalized RSV values rather than absolute search counts, meaning RSV reflects the relative popularity of a search term within a given location and time period and should not be interpreted as a direct measure of disease burden, environmental exposure, or public awareness. Because RSV values are independently normalized, direct comparisons between heterogeneous search terms should be interpreted with caution.

Third, disparities in internet access, digital literacy, socioeconomic status, age, education, and geographic location introduce a systematic digital divide, whereby populations with limited online access — including those most vulnerable to AP-related CVD — are underrepresented in search data. Consequently, findings may not fully represent information-seeking behaviour within the broader population.

Fourth, the high proportion of missing or insufficient data across countries may reflect low search volumes, limitations in keyword selection, language restrictions, or structural barriers to digital engagement. The restriction to English-language terms likely underrepresents search behavior in non-English-speaking populations, including regions with the highest AP and CVD burden.

Fifth, although partial correlation analysis controlling for mask was conducted as a sensitivity analysis to address pandemic confounding, this approach provides only an approximation of pandemic-free associations, as mask search behavior does not fully capture the breadth of COVID-19-related confounding across all terms.

Sixth, search behavior may be influenced by short-term media coverage, public health campaigns, or acute events, leading to transient spikes that do not necessarily reflect sustained information-seeking behavior. Google Trends data are inherently ecological and cannot be used to infer causation or measure information-seeking behavior at the individual level.

Seventh, although time-series methods including ARIMAX and Granger causality analysis were applied to explore temporal and predictive relationships, these approaches rely on assumptions of stationarity and may be sensitive to model specification and lag selection. The absence of significant Granger causality does not rule out real-world associations but rather indicates limited predictive power within the observed search data.

Finally, it should be explicitly acknowledged that the general public's perception of the cardiovascular effects of air pollution is likely to be biased or incomplete. Online information-seeking behavior reflects lay understanding rather than evidence-based knowledge, and search queries may be driven by misinformation, media framing, or symptom-focused concerns rather than accurate comprehension of AP–CVD pathways.

Therefore, findings should be interpreted as indicators of relative online information-seeking behavior rather than precise measures of population-level environmental health literacy, cardiovascular disease burden, or AP exposure.

## 6 Conclusion

This infodemiological analysis of global Google Trends data reveals a notable disparity between the scientific consensus on AP as a CVD risk factor and online information-seeking behavior related to this association. These findings underscore the need for communication strategies that translate technical AP indicators into tangible cardiovascular health outcomes, alongside digital surveillance tools and policy interventions that bridge the gap between environmental exposure evidence and public health action.

## Data Availability

Publicly available datasets were analyzed in this study. This data can be found here: https://trends.google.com/trends/explore?geo=PKhl=en-GB.
