# Supplementary material for: Global information-seeking behavior of air pollution and cardiovascular disease: insights from google trends analysis
Source: Front Epidemiol. 2026 Jul 8;6:1874654. doi: 10.3389/fepid.2026.1874654 (PMC13388820; doi:10.3389/fepid.2026.1874654)
Supplement: Supplementary file 1 [file Table1.docx]

***Supplementary Table 1: Country-level relative search volume (RSV) for AP and CVD-related search terms across 27 overlap countries (June 2020–June 2025).***

| **Country** | **Air pollution** | **Environmental Pollution** | **PM2.5** | **AQI** | **Mask** | **Heart Disease** | **Heart Attack** | **High Blood Pressure** | **Chest Pain** | **Mean RSV** |
| --- | --- | --- | --- | --- | --- | --- | --- | --- | --- | --- |
| Egypt | 3 | 1 | 3 | 0 | 10 | 10 | 19 | 2 | 5 | 6.3 |
| Saudi Arabia | 3 | 1 | 3 | 0 | 16 | 11 | 7 | 4 | 9 | 6.4 |
| Sweden | 3 | 0.5 | 11 | 0 | 37 | 3 | 3 | 1 | 3 | 6.95 |
| South Korea | 10 | 1 | 24 | 2 | 11 | 5 | 3 | 2 | 2 | 7.4 |
| Taiwan | 7 | 0.5 | 54 | 0 | 8 | 3 | 0 | 1 | 0 | 8.15 |
| Bangladesh | 17 | 2 | 5 | 3 | 18 | 14 | 15 | 5 | 10 | 10.3 |
| Thailand | 7 | 0.5 | 72 | 2 | 16 | 7 | 1 | 1 | 2 | 11.55 |
| Tanzania | 32 | 5 | 0 | 0 | 14 | 39 | 0 | 10 | 0 | 12.9 |
| Sri Lanka | 25 | 4 | 0 | 15 | 29 | 23 | 35 | 9 | 21 | 17.9 |
| Malaysia | 17 | 2 | 16 | 3 | 57 | 23 | 20 | 11 | 16 | 18.6 |
| United Arab Emirates | 25 | 1 | 14 | 12 | 46 | 18 | 31 | 15 | 34 | 21.7 |
| Hong Kong | 21 | 2 | 60 | 7 | 71 | 16 | 7 | 5 | 7 | 22.2 |
| Pakistan | 35 | 3 | 8 | 24 | 25 | 28 | 38 | 19 | 33 | 23.3 |
| India | 78 | 6 | 6 | 17 | 28 | 18 | 34 | 7 | 27 | 23.7 |
| Ethiopia | 50 | 6 | 0 | 0 | 18 | 84 | 0 | 13 | 0 | 24.9 |
| South Africa | 35 | 5 | 4 | 2 | 31 | 23 | 39 | 33 | 59 | 25 |
| Nigeria | 35 | 8 | 3 | 0 | 14 | 40 | 24 | 33 | 70 | 27.7 |
| Ireland | 10 | 0 | 14 | 0 | 65 | 27 | 46 | 28 | 63 | 28 |
| New Zealand | 10 | 0 | 36 | 0 | 74 | 28 | 45 | 22 | 44 | 29 |
| Canada | 14 | 1 | 27 | 33 | 66 | 24 | 49 | 23 | 41 | 30.4 |
| Ghana | 60 | 8 | 0 | 0 | 20 | 48 | 29 | 35 | 57 | 31.6 |
| Zambia | 50 | 6 | 0 | 0 | 16 | 100 | 0 | 36 | 68 | 31.8 |
| Kenya | 42 | 7 | 9 | 0 | 20 | 53 | 35 | 39 | 70 | 32.2 |
| Singapore | 25 | 2 | 37 | 0 | 100 | 28 | 42 | 26 | 38 | 32.8 |
| Nepal | 100 | 10 | 12 | 20 | 30 | 41 | 37 | 17 | 40 | 34 |
| Australia | 17 | 1 | 46 | 6 | 88 | 34 | 47 | 22 | 47 | 35.1 |
| Philippines | 50 | 6 | 10 | 4 | 55 | 71 | 50 | 33 | 48 | 40.5 |
