# Supplementary material for: Global information-seeking behavior of air pollution and cardiovascular disease: insights from google trends analysis
Source: Front Epidemiol. 2026 Jul 8;6:1874654. doi: 10.3389/fepid.2026.1874654 (PMC13388820; doi:10.3389/fepid.2026.1874654)

***Supplementary Figure 1.*** *Heatmap of Relative Search Volume (RSV) for air pollution and cardiovascular disease–related search terms across selected cities (2020–2025). Darker shades of red indicate higher search interest, while lighter shades represent lower RSV values.*


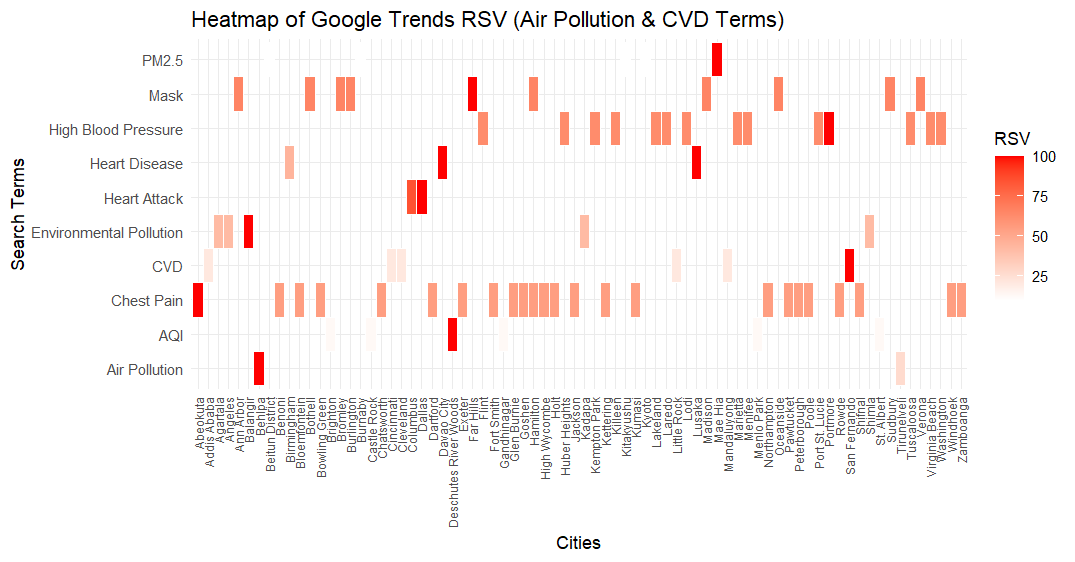


***Supplementary Figure 2:*** *Graphical Presentation of Air Pollution and Cardiovascular Disease: Insights from Global Trend Analysis*


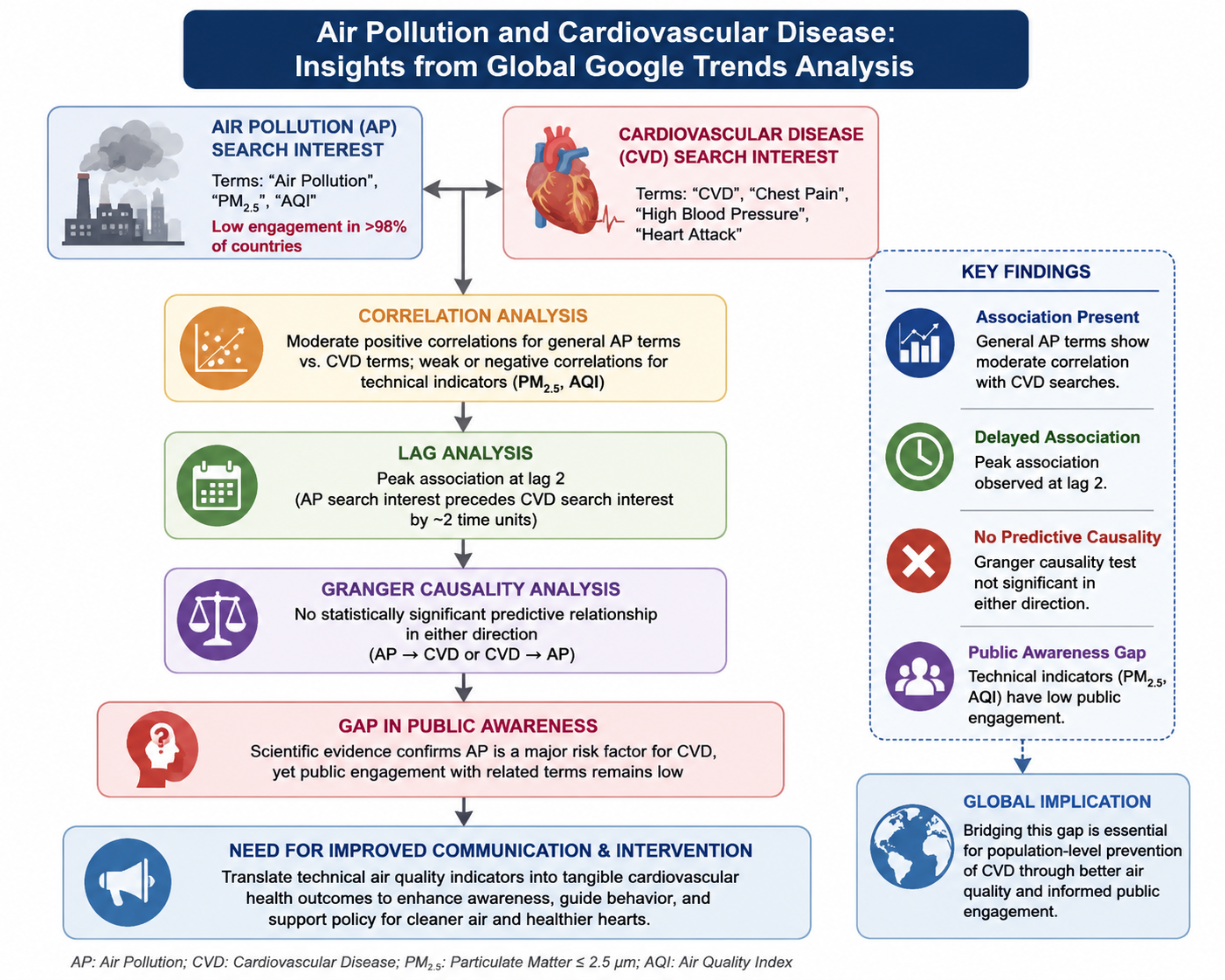


***Supplementary Figure 3:*** *Lagged association between air pollution and cardiovascular disease search interest*


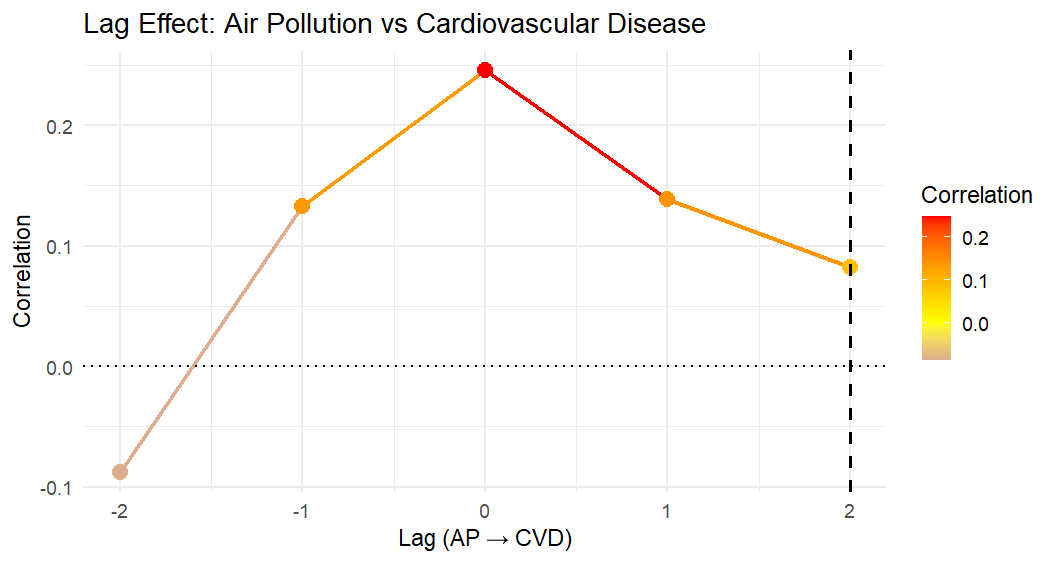

Supplement: Supplementary file 2 [file Table2.docx]
